# The effect of argatroban on early neurological deterioration and outcomes in minor ischemic stroke: preliminary findings

**DOI:** 10.3389/fneur.2024.1363358

**Published:** 2024-03-08

**Authors:** Xuehong Jin, Xia Li, Hong Zhang, Xiaohan Yao, Yongquan Gu, Shaofang Pei, Lan Hu

**Affiliations:** ^1^Department of Neurology, Suzhou Municipal Hospital, Nanjing Medical University, Suzhou, China; ^2^Department of Neurology, Suzhou Ninth People’s Hospital, Soochow University, Suzhou, China

**Keywords:** argatroban, early neurological deterioration (END), minor ischemic stroke (MIS), activated partial thrombin time (APTT), prognosis, functional outcome

## Abstract

**Background:**

Minor ischemic stroke (MIS) is associated with early neurological deterioration (END) and poor prognosis. Here, we investigated whether argatroban administration can mitigate MIS-associated END and improve functional outcomes by monitoring activated partial thrombin time (APTT).

**Methods:**

Data were collected for patients with MIS admitted to our hospital from January 2019 to December 2022. Patients were divided into a dual antiplatelet therapy (DAPT) group (aspirin + clopidogrel) and an argatroban group (aspirin + argatroban). Those in the latter group who achieved a target APTT of 1.5–3-fold that of baseline and <100 s at 2 h after argatroban infusion were included in the argatroban subgroup. The primary outcome was the END rate of the DAPT group versus that of the argatroban group or the argatroban subgroup. Secondary outcomes included the proportion of patients with modified Rankin Scale (mRS) 0–2 at 7 and 90 days. In addition, baseline date were compared between patients with and without END in the argatroban group.

**Results:**

363 patients were included in the DAPT group and 270 in the argatroban group. There were no significant differences in any above outcome between them. 207 pairs were included in the DAPT group and the argatroban subgroup after 1:1 propensity score matching (PSM). Significant differences were observed in the proportion of END (OR, 2.337; 95% CI, 1.200–4.550, *p* = 0.011) and mRS 0–2 at 7 days (OR, 0.624; 95% CI, 0.415–0.939, *p* = 0.023), but not in mRS 0–2 at 90 days or the hemorrhagic events between the two groups. In the argatroban group, univariate analysis showed that the rate of diabetes (OR, 2.316; 95% CI, 1.107–4.482, *p* = 0.023), initial random blood glucose (OR, 1.235; 95% CI, 1.070–1.425, *p* = 0.004), drinking history (OR, 0.445; 95% CI, 0.210–0.940, *p* = 0.031) or those reaching the target APTT (OR, 0.418; 95% CI, 0.184–0.949, *p* = 0.033) was significantly different among patients with and without END. However, there were no statistical differences in these parameters between them following multivariate analysis.

**Conclusion:**

In patients with MIS, argatroban administration and reaching the target APTT can reduce the incidence of END and improve short-term functional prognosis.

## 1 Introduction

Minor ischemic stroke (MIS) is common and accounts for 44.0 and 51.2% of all ischemic strokes according to two China National Stroke Registries (1). The results of the CHANCE trial suggested that dual therapy with aspirin and clopidogrel can significantly reduce the risk of 90 days stroke recurrence in patients with transient ischemic attack (TIA) and MIS, and this regimen is currently recommended in the guidelines for healthcare professionals from the American Heart Association/American Stroke Association (2, 3). However, in addition to the high risk of recurrence within 3 months, early neurological deterioration (END) is also a common occurrence in patients with MIS. It has been reported that the incidence of END in patients with MIS is as high as 21 to 50% (4–6). END not only increases the difficulty of treatment in the acute phase of MIS, but is also associated with poor prognosis (disability, dysfunction, or death). Approximately one-third of patients with MIS experience permanent dysfunction (7–9).

In addition to antiplatelet aggregation therapy, the use of anticoagulant therapy in the treatment of acute ischemic stroke (AIS) is currently also the focus of intensive research efforts. However, most anticoagulants are not currently recommended for the early treatment of AIS owing to concerns relating to bleeding risks. A systematic search involving 14 clinical trials found no evidence to support the early use of heparin, heparinoids, or low-molecular-weight heparin after stroke, and there was also no consistent evidence for warfarin or direct oral anticoagulants. Argatroban was found to be the only anticoagulant that achieved significant positive results in the early stage of large-artery ischemic stroke (10).

Argatroban, a direct thrombin inhibitor, has an immediate anticoagulant effect after intravenous administration. It not only can greatly prolong activated partial thrombin time (APTT), prothrombin time, and other coagulation factors at different doses but also has a half-life of drug clearance is 39 to 51 min, and APTT will return to normal within 2 to 4 h after drug withdrawal. Therefore, the infusion rate of drugs can be monitored and regulated by APTT in clinic. Its main contraindications includes patients with hemorrhagic disorders, cardiogenic cerebral embolism, and patients with hepatic insufficiency. At present, argatroban has been approved for the treatment of AIS in China, Japan, South Korea, and a few other countries. In china, it was officially listed in 2002 and approved by National Medical Products Administration in 2005 for AIS patients within 48 h of onset.

Here, based on APTT monitoring, we investigated whether the administration of argatroban plus aspirin can reduce the incidence of END and improve prognosis in patients with MIS compared with dual antiplatelet therapy (DAPT).

## 2 Materials and methods

### 2.1 Study design and patients

This was a retrospective, observational, cohort study involving data from the stroke database of the Affiliated Suzhou Hospital of Nanjing Medical University (Suzhou Municipal Hospital), obtained by neurologists using face-to-face assessments or telephone follow-up. Data for patients with MIS were continuously collected from January 2019 to December 2022. This study was approved by the Ethics Committee of Suzhou Municipal Hospital (approval number: KL901121), Patients were consented by an informed consent process that was reviewed by the Ethics Committee of Suzhou Municipal Hospital.

The inclusion criteria were as follows: (i) arrival within 24 h of the last known normal; (ii) an initial National Institutes of Health Stroke Scale (NIHSS) score of five or less; (iii) the patients were treated with one of two regimens, namely, aspirin 100 mg/day combined with clopidogrel 75 mg/day, clopidogrel loading dose 300 mg; or aspirin 100 mg/day combined with argatroban, in which argatroban 60 mg/d was continuously pumped for 48 h, followed by 10 mg infusion over 3 h twice daily for 5 days. The total treatment time for all the patients was not less than 7 days. Patients were excluded based on the following criteria: (i) experienced cardiogenic stroke; (ii) were intravenous thrombolysis; (iii) were pregnant or had malignant tumors, lower respiratory tract infections, heart failure, or severe anemia; and (iv) their follow-up records were incomplete.

The enrolled patients who received argatroban plus aspirin were assigned to the argatroban group and those treated with aspirin combined with clopidogrel were assigned to the dual antiplatelet therapy (DAPT) group. Subsequently, patients from the argatroban group whose APTT values were 1.5- to 3.0-fold those at baseline and less than 100 s 2 h after argatroban infusion were included in the argatroban subgroup.

### 2.2 Clinical and laboratory data collection

The following information was gathered for each patient from our database: baseline demographic data (age and gender); risk factors for stroke, including hypertension, diabetes mellitus, hyperlipidemia, previous stroke or TIA, and smoking or drinking history; laboratory results, such as initial random blood glucose (RBG), triglyceride (TG), total cholesterol (TC), low-density lipoprotein cholesterol (LDL-C), and APTT; clinical indexes, including initial blood pressure, time from onset to admission, NIHSS score for each of the 7 days, END or not, and modified Rankin Scale (mRS) score at 7 and 90 days after hospitalization. Data for bleeding events (e.g., intracranial, gastrointestinal, urinary tract, and skin mucosal hemorrhaging) were also collected.

APTT was detected by the CS-2100i coagulation analyzers (Sysmex Corporation, Japan), and the testing procedure was as follows: (i) blood samples (2.7 mL) and 3.8%(w/v) sodium citrate (0.3 mL) were mixed, and were centrifuged (3,000 r/min × 15 min) after collection at room temperature; (ii) 50 μL citrated plasma sample and 50 μL APTT reagent were mixed in a preheated test tube and incubated at 37°C for 3 min. Thereafter, 50 μL calcium chloride solution was added; (iii) the sample was illuminated with light with a wavelength of 660 nm, and the turbidity of the plasma during coagulation can be determined by measuring the change in the intensity of the scattered light; (iv) APTT can be obtained from the coagulation curve.

### 2.3 The definition of MIS, END, and favorable functional outcome

MIS referred to patients with an initial NIHSS score of 0–5 and a score of 0 or 1 for each baseline NIHSS score item (items 1a to 1c being 0) (11). END was defined as an incremental increase in the NIHSS score of ≥1 point in motor power or ≥2 points in the total score within 7 days of admission (12). Favorable functional outcome was defined as functional independence (mRS score of 0–2).

In addition, major bleeding was defined as follows: (i) fatal bleeding, and/or (ii) symptomatic bleeding in a critical area or organ, such as intracranial, intraspinal, intraocular, retroperitoneal, intraarticular or pericardial, or intramuscular with compartment syndrome, and/or (iii) bleeding causing a fall in hemoglobin level of 20 g/L^−1^ (1.24 mmol L^−1^) or more, or leading to transfusion of two or more units of whole blood or red cells (13).

### 2.4 Outcomes

The primary outcome was the incidence of END within 7 days after admission in patients of the DAPT group versus those of the argatroban group or the argatroban subgroup. Secondary outcomes included the proportion of patients with an mRS score of 0–2 at 7 and 90 days. The safety outcome was the incidence of hemorrhagic events, including intracranial, gastrointestinal, urinary tract, and skin mucosal hemorrhaging. In addition, baseline characteristics were compared between patients with and without END in the argatroban group.

### 2.5 Statistical analysis

Data were analyzed using SPSS 23.0 for Windows. The mean ± SD or median (interquartile range) was used to describe continuous variables depending on the normality of the distribution and were compared using the independent samples *T*-test or Mann–Whitney U test. Categorical variables are presented as frequency and were compared by Pearson’s *χ*^2^ test. Differences in baseline characteristics between patients in the argatroban subgroup and the DAPT group were adjusted with 1:1 propensity score matching (PSM) using the nearest-neighbor matching algorithm with a caliper width of 0.2. Univariate and multivariate logistic regression analyses were performed to describe the differences between patients with and those without END in the argatroban group. All tests of significance were two-tailed and *p* < 0.05 was considered significant.

## 3 Results

### 3.1 Baseline characteristics and outcome assessment

**Figure 1:**
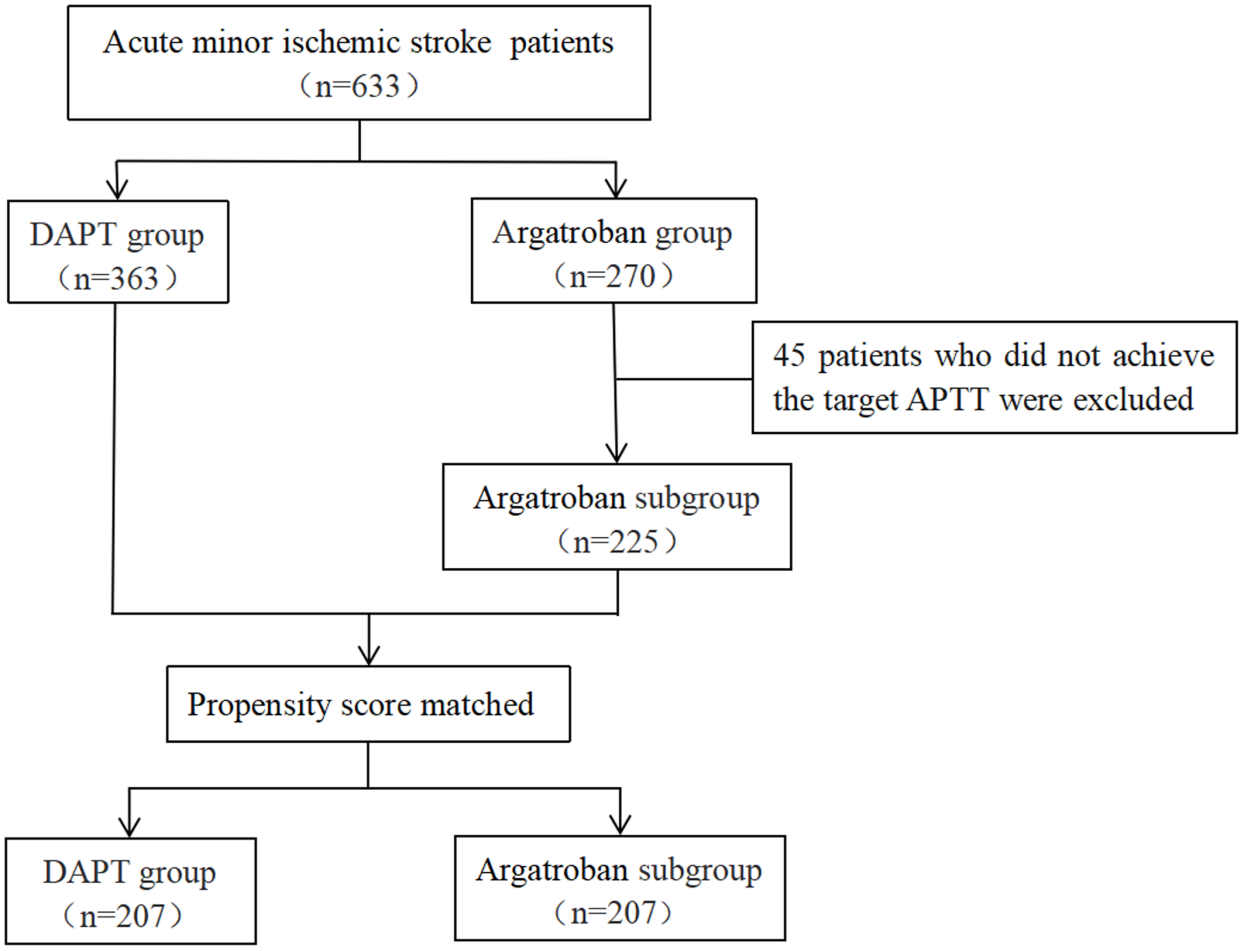
Study flow chart of the patient selection process.

As shown in Figure 1 and Table 1, a total of 633 patients were enrolled in our study, including 363 in the DAPT group and 270 in the argatroban group. Overall, 87 patients (13.7%) experienced END, and 52 (8.2%) experienced bleeding events, including intracranial, gastrointestinal, urinary tract, and skin mucosal hemorrhage; however, no major bleeding occurred. The analyzed baseline characteristics, including age, sex, risk factors (hypertension, diabetes, hyperlipidemia, previous stroke or TIA, smoking, and drinking history), and clinical and laboratory findings (initial blood glucose, TG, TC, LDL-C, APTT, baseline SBP and DBP, time from onset to admission, and NIHSS score on admission) were similar between the two groups. In total, 53 patients (14.6%) had END and 28 (7.7%) experienced bleeding events in the DAPT group, compared with 34 (12.6%) and 24 (8.9%) in the argatroban group, respectively. There were no significant differences in the proportion of END and bleeding events between the two groups (*p* > 0.05). Similarly, no significant differences in secondary outcome parameters (proportion of mRS score 0–2 at 7 and 90 days) were detected between the two groups (*p* > 0.05).

**Table 1:** Baseline characteristics and outcomes in the DAPT and argatroban groups.

	DAPT group (*n* = 363)	Argatroban group (*n* = 270)	Statistical value	*p*-value
Age, years, median (IQR)	67 (58–75)	68 (61–76)	−1.553	0.120
Female, *n* (%)	246 (67.8)	177 (65.6)	0.342	0.559
Hypertension, *n* (%)	212 (58.4)	138 (51.1)	3.330	0.068
Diabetes mellitus, *n* (%)	82 (22.6)	75 (27.8)	2.235	0.135
Hyperlipidemia, *n* (%)	51 (14.0)	41 (15.2)	0.161	0.688
Previous stroke or TIA, *n* (%)	50 (13.8)	30 (11.1)	0.994	0.319
Smoking, *n* (%)	126 (34.7)	76 (28.1)	3.069	0.080
Drinking, *n* (%)	190 (52.3)	142 (52.6)	0.004	0.950
Initial RBG, mmol/L, median (IQR)	5.7 (5.01–6.71)	5.44 (4.77–6.57)	−1.606	0.108
TG, mmol/L, median (IQR)	1.25 (1.01–1.83)	1.40 (1.02–1.70)	−0.566	0.572
TC, mmol/L, median (IQR)	4.80 (4.06–5.70)	4.68 (3.80–5.60)	−1.779	0.075
LDL-C, mmol/L, median (IQR)	2.83 (2.09–3.36)	2.81 (2.11–3.50)	−0.505	0.614
Initial APTT, s, median (IQR)	25.60 (22.20–27.60)	25.90 (23.10–27.93)	−1.903	0.057
Initial SBP, mmHg, median (IQR)	141 (128–158)	139 (128.5–153.0)	−1.320	0.187
Initial DBP, mmHg, median (IQR)	78 (69–87)	78 (68–87)	−0.439	0.661
Time from onset to admission, min, median (IQR)	420 (308–600)	406 (300–540)	−1.603	0.109
Initial NIHSS, median (IQR)	3 (2–4)	3 (2–5)	−1.603	0.109
END, *n* (%)	53 (14.6)	34 (12.6)	0.527	0.468
mRS 0–2 at 7 days, *n* (%)	221 (60.9)	180 (66.7)	2.232	0.135
mRS 0–2 at 90 days, *n* (%)	288 (79.3)	219 (81.1)	0.305	0.581
Intracranial hemorrhage, *n* (%)	4 (1.1)	4 (1.5)	0.179	0.672
Skin mucosal hemorrhage, *n* (%)	8 (2.2)	6 (2.2)	0.000	0.988
Urinary tract hemorrhage, *n* (%)	4 (1.1)	4 (1.5)	0.179	0.672
Gastrointestinal hemorrhage, *n* (%)	12 (3.3)	10 (3.7)	0.073	0.787

DAPT, dual antiplatelet therapy; TIA, transient ischemic attack; RBG, random blood glucose; TG, triglyceride; TC, total cholesterol; LDL-C, low-density lipoprotein cholesterol; APTT, activated partial thrombin time; SBP, systolic blood pressure; DBP, diastolic blood pressure; NIHSS, National Institutes of Health Stroke Scale; END, early neurological deterioration; mRS, modified Rankin Scale.

### 3.2 Subgroup analysis after PSM

In total, 43 patients had APTT values more than 1.5-fold lower than those at baseline after the 2 h argatroban infusion and 2 patients had APTT values more than 3-fold higher than those at baseline; however, no patient had APTT longer than 100 s. A total of 225 patients were enrolled in the argatroban subgroup. As shown in Table 2, most baseline data were balanced between the DAPT group and the argatroban subgroup, except for initial RBG and APTT. After 1:1 PSM, 207 pairs of patients were included, and all baseline characteristics, including age, sex, risk factors, and clinical and laboratory findings were balanced between the two groups.

**Table 2:** Baseline characteristics of patients in the DAPT group and the argatroban subgroup before and after PSM.

	Before PSM				After PSM			
	DAPT group (*n* = 363)	Argatroban subgroup (*n* = 225)	Statistical value	*p*-value	DAPT group (*n* = 207)	Argatroban subgroup (*n* = 207)	Statistical value	*p*-value
Age, years, median (IQR)	67 (58–75)	67 (60.5–76)	−1.018	0.309	69 (58–76)	67 (61–75)	−0.032	0.974
Females, *n* (%)	246 (67.8)	145 (64.4)	0.689	0.407	133 (64.3)	136 (65.7)	0.096	0.757
Hypertension, *n* (%)	212 (58.4)	114 (50.7)	3.365	0.067	120 (58.0)	110 (53.1)	0.978	0.323
Diabetes mellitus, *n* (%)	82 (22.6)	54 (24.0)	0.155	0.693	56 (27.1)	46 (22.2)	1.301	0.254
Hyperlipidemia, *n* (%)	51 (14.0)	36 (16.0)	0.419	0.517	27 (13.0)	30 (14.5)	0.183	0.669
Previous stroke or TIA, *n* (%)	50 (13.8)	22 (9.8)	2.064	0.151	23 (11.1)	22 (10.6)	0.025	0.875
Smoking, *n* (%)	126 (34.7)	63 (28.0)	2.868	0.090	72 (34.8)	61 (29.5)	1.340	0.247
Drinking, *n* (%)	190 (52.3)	123 (54.7)	0.302	0.583	99 (47.8)	115 (55.6)	2.476	0.116
Initial RBG, mmol/L, median (IQR)	5.70 (5.01–6.71)	5.20 (4.63–5.88)	−5.619	0.000	5.25 (4.63–5.97)	5.25 (4.72–5.93)	−0.466	0.641
TG, mmol/L, median (IQR)	1.25 (1.01–1.83)	1.40 (1.01–1.70)	−0.576	0.564	1.21 (0.99–1.81)	1.40 (1.07–1.70)	−1.323	0.186
TC, mmol/L, median (IQR)	4.80 (4.06–5.70)	4.70 (3.81–5.52)	−1.616	0.106	4.56 (3.71–5.34)	4.66 (3.80–5.40)	−0.755	0.450
LDL-C, mmol/L, median (IQR)	2.83 (2.09–3.36)	2.80 (2.11–5.50)	−0.620	0.535	2.73 (2.04–3.29)	2.78 (2.10–3.50)	−0.847	0.397
APTT, s, median (IQR)	25.60 (22.20–27.60)	25.70 (23.00–28.05)	−2.007	0.045	25.5 (22.30–27.80)	25.70 (22.90–28.10)	−0.568	0.117
Initial SBP, mmHg, median (IQR)	141 (128–158)	139 (127–154)	−1.076	0.282	139 (127–154)	140 (130–154)	−0.632	0.527
Initial DBP, mmHg, median (IQR)	78 (69–87)	78 (68–87)	−0.370	0.712	79 (70–87)	78 (69–87)	−0.036	0.971
Time from onset to admission, min, median (IQR)	420 (308–600)	406 (300–556)	−1.226	0.220	395 (300–562)	420 (320–578)	−0.872	0.383
Initial NIHSS, median (IQR)	3 (2–4)	3(2–5)	−1.479	0.139	3 (2–4)	2 (1–4)	−0.195	0.846

DAPT, dual antiplatelet therapy; PSM, propensity score matching; TIA, transient ischemic attack; RBG, random blood glucose; TG, triglyceride; TC, total cholesterol; LDL-C, low-density lipoprotein cholesterol; APTT, activated partial thrombin time; SBP, systolic blood pressure; DBP, diastolic blood pressure; NIHSS, National Institutes of Health Stroke Scale; END, early neurological deterioration; mRS, modified Rankin Scale.

**Figure 2:**
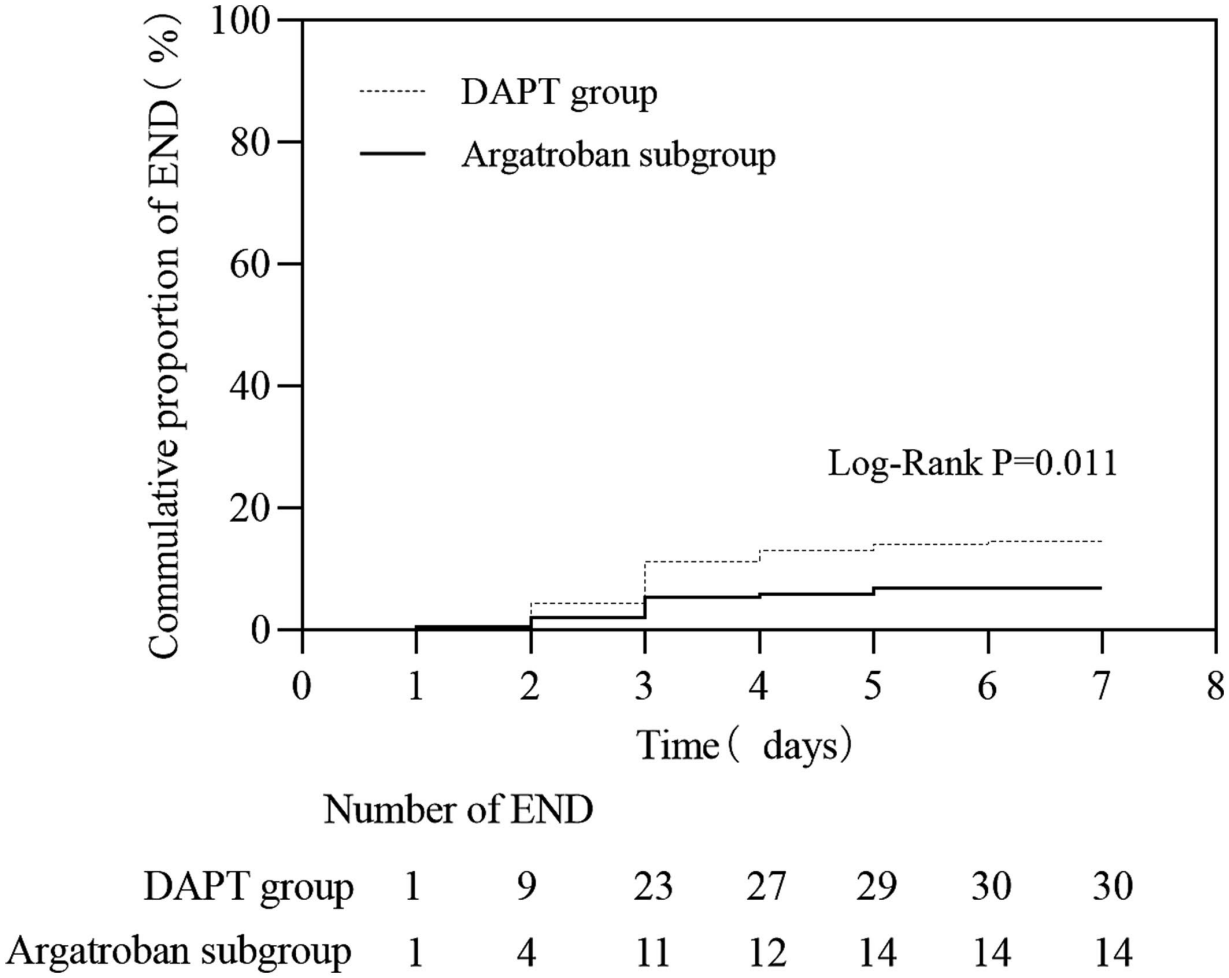
The early neurological deterioration (END) rate within 7 days in matched patients between the DAPT group and the argatroban group.

Among the matched populations, 30 patients (10.1%) experienced END, and 17 (8.2%) had bleeding events in the DAPT group, while in the argatroban subgroup, 14 (6.8%) and 21 (9.9%) patients experienced END and bleeding events, respectively (Table 3). The proportion of patients with END (OR, 2.337; 95% CI, 1.200–4.550, *p* = 0.011) differed significantly between the DAPT group and the argatroban subgroup; in contrast, no significant difference in the incidence of bleeding events was observed between these two treatment groups (*p* > 0.05). As shown in Figure 2, the END rate in both groups gradually increased within 7 days, with most instances occurring in the first 72 h (23 vs. 11, respectively), while the cumulative END rate was significantly different between the DAPT group and the argatroban subgroup (10.1% vs. 6.8%, *p* = 0.011).

**Table 3:** Comparative analysis of matched patients of the DAPT group and argatroban subgroup.

	DAPT group (*n* = 207)	Argatroban subgroup (*n* = 207)	*p*-value	OR (95% CI)
Primary outcome				
END, *n* (%)	30 (14.5)	14 (6.8)	0.011	2.337 (1.200–4.550)
Secondary outcome				
mRS 0–2 at 7 days, *n* (%)	124(59.9)	146(70.5)	0.023	0.624 (0.415–0.939)
mRS 0–2 at 90 days, *n* (%)	163(78.7)	171(82.6)	0.319	0.780 (0.478–1.773)
Safety outcome				
Intracranial hemorrhage, *n* (%)	3 (1.4)	4 (1.9)	0.703	0.746 (0.165–3.377)
Skin mucosal hemorrhage, *n* (%)	6 (2.9)	5 (2.4)	0.760	1.206 (0.362–4.015)
Urinary tract hemorrhage, *n* (%)	3 (1.4)	4 (1.9)	0.703	0.746 (0.165–3.377)
Gastrointestinal hemorrhage, *n* (%)	5 (2.4)	8 (3.9)	0.398	0.616 (0.198–1.914)

END, early neurological deterioration; mRS, modified Rankin Scale.

### 3.3 Evaluation of short-term and long-term functional prognosis

**Figure 3:**
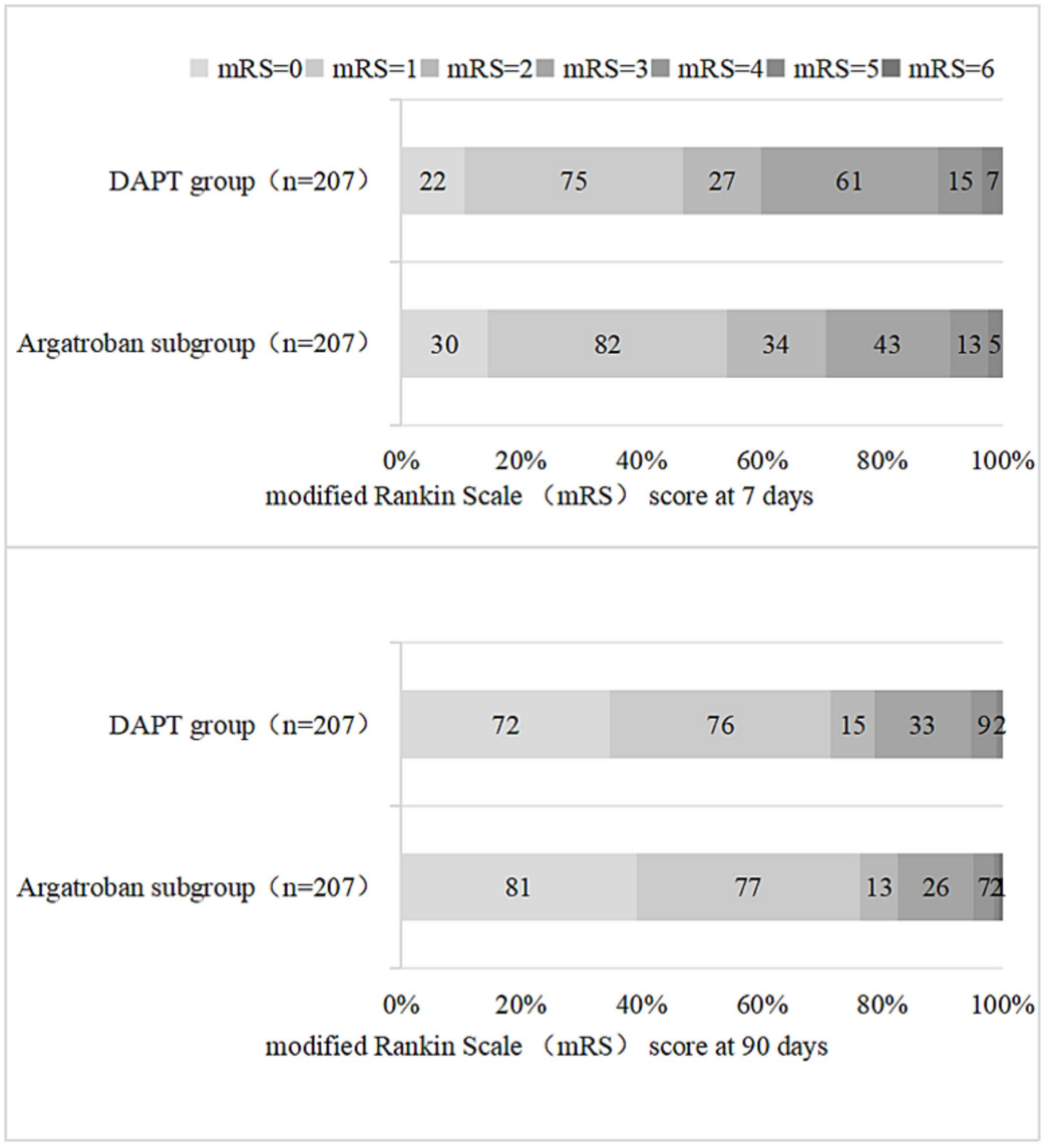
Distribution of the modified Rankin Scale (mRS) scores in the DAPT group and the argatroban group at 7 and 90 days.

As shown in Table 3 and Figure 3, a total of 124 (59.9%) and 163 (78.7%) patients in the DAPT group and 146 (70.5%) and 171 (82.6%) patients in the argatroban subgroup achieved good functional outcomes (mRS score 0–2) at 7 and 90 days, respectively. The proportion of patients with a favorable functional outcome at 7 days was significantly higher in the argatroban subgroup than in the DAPT group (OR, 0.624; 95% CI, 0.415–0.939, *p* = 0.023), whereas no significant difference in this parameter was observed between the two groups at 90 days (*p* > 0.05).

### 3.4 Comparative analysis between patients with and those without END in the argatroban group

Univariate logistic regression analysis showed that the proportion of patients with diabetes (OR, 2.316; 95% CI, 1.107–4.482, *p* = 0.023) was significantly higher in the END than in the non-END group; meanwhile, initial RBG (OR, 1.235; 95% CI, 1.070–1.425, *p* = 0.004), the proportion of patients with a drinking history (OR, 0.445; 95% CI, 0.210–0.940, *p* = 0.031), and the proportion of patients reaching the target APTT (OR, 0.418; 95% CI, 0.184–0.949, *p* = 0.033) were also significantly higher in the END group. However, further multivariate logistic regression analysis showed that there was no statistical difference in any of these parameters between the two groups (Table 4).

**Table 4:** Univariate and multivariate analysis between patients with END and those without END in the argatroban group.

	Patients without END (*n* = 236)	Patients with END (*n* = 34)	Univariate analysis		Multivariate analysis	
			*p*-value	OR (95% CI)	*p*-value	OR (95% CI)
Age, years, median (IQR)	67.5 (61–76)	69 (60–78.8)	0.759	1.005 (0.973–1.039)		
Female, *n* (%)	151 (76.5)	26 (64.0)	0.152	1.829 (0.793–4.220)		
Hypertension, *n* (%)	118 (50.0)	20 (58.8)	0.336	1.429 (0.698–2.962)		
Diabetes mellitus, *n* (%)	60 (25.4)	15 (44.1)	0.023	2.316 (1.107–4.482)	0.293	1.140 (0.893–1.457)
Hyperlipidemia, *n* (%)	37 (27.2)	4 (11.8)	0.060	0.357 (0.118–1.082)	0.928	1.056 (0.326–3.417)
Previous stroke or TIA, *n* (%)	24 (17.6)	6 (17.6)	1.000	1.000 (0.373–2.680)		
Smoking, *n* (%)	67 (46.3)	9 (26.5)	0.017	0.371 (0.161–0.853)	0.988	0.993 (0.405–2.438)
Drinking, *n* (%)	130 (55.1)	12 (35.3)	0.031	0.445 (0.210–0.940)	0.087	2.042 (0.902–4.622)
Initial RBG, mmol/L, median (IQR)	5.29 (4.74–2.11)	5.25 (4.47–9.03)	0.004	1.235 (1.070–1.425)	0.293	1.140 (0.893–1.457)
TG, mmol/L, median (IQR)	1.4 (1.01–1.70)	1.35 (1.07–1.56)	0.747	0.925 (0.576–1.484)		
TC, mmol/L, median (IQR)	4.63 (3.8–5.59)	5.0 (3.97–5.88)	0.261	1.169 (0.890–1.536)		
LDL-C, mmol/L, median (IQR)	2.80 (2.10–3.50)	2.88 (2.28–3.50)	0.685	1.079 (0.747–1.559)		
APTT, s, median (IQR)	25.80 (23.10–28.20)	25.55 (22.25–26.98)	0.177	0.937 (0.852–1.030)		
Initial SBP, mmHg, median (IQR)	139 (129–153.6)	135.5 (125.3–146)	0.118	0.984 (0.964–1.004)		
Initial DBP, mmHg, median (IQR)	78 (68–87.8)	77 (66.8–82.3)	0.182	0.981 (0.954–1.009)		
Time from onset to admission, min, median (IQR)	405.5 (300–546)	415 (321–540)	0.920	1.000 (0.998–1.002)		
Initial NIHSS, median (IQR)	3 (2–5)	3 (1–4)	0.321	0.886 (0.696–1.126)		
Achieved target APTT, *n* (%)	201 (85.2)	24 (70.6)	0.033	0.418 (0.184–0.949)	0.064	0.278 (0.071–1.080)

DAPT, dual antiplatelet therapy; PSM, propensity score matching; TIA, transient ischemic attack; RBG, random blood glucose; TG, triglyceride; TC, total cholesterol; LDL-C, low-density lipoprotein cholesterol; APTT, activated partial thrombin time; SBP, systolic blood pressure; DBP, diastolic blood pressure; NIHSS, National Institutes of Health Stroke Scale; END, early neurological deterioration; mRS, modified Rankin Scale.

## 4 Discussion

Argatroban is a derivative of arginine and exerts its anticoagulant effects primarily by inhibiting reactions catalyzed or induced by thrombin, including fibrin formation, the activation of clotting factors, the activation of protease C, and platelet aggregation (14–16). In addition, animal trials have shown that argatroban provides a synergistic neuroprotective effect by decreasing C5a receptor synthesis and expression in the brain, reducing inflammatory cytokine levels, mitigating brain edema, and inhibiting nerve cell apoptosis (17).

Our study found no statistical difference between the argatroban and DAPT groups in terms of reducing END rate and improving functional outcomes. However, the proportion of END and favorable functional outcome at 7 days in the argatroban subgroup were superior to those in the DAPT group, indicating that focusing on the target APTT may be an important point for studying the clinical efficacy of argatroban.

A multi-center, randomized, double-blind, placebo-controlled study conducted in Japan in 1997 was the first to demonstrate the efficacy and safety of argatroban in the treatment of AIS caused by large artery atherosclerosis (15). More studies on the efficacy of argatroban in AIS have subsequently been undertaken. One clinical study found that argatroban increased regional basal vein of Rosenthal drainage in the ipsilateral hemisphere of the brains of patients with acute paraventricular ischemic stroke caused by intracranial large artery atherosclerosis; improved the NIHSS score on day 7 after stroke onset; and increased the proportion of patients with mRS scores of 0–1 on day 90 (18). Moreover, compared with aspirin alone, aspirin combined with agatroban can significantly reduce the levels of serum fibrinogen and neuropeptide Y in patients with AIS combined with END, as well as improve neurological impairment (19). A meta-analysis showed that, compared with the control treatment, argatroban infusion significantly ameliorated neurological function in patients with ischemic stroke, including improving NIHSS and the modified Barthel index scores, and decreasing the incidence of END (20). A largest randomized clinical trial showed among patients with AIS with END, treatment with argatroban and antiplatelet therapy resulted in a better functional outcome at 90 days in both unadjusted and adjusted analyses (21).

There are relatively few studies on argatroban in the treatment of MIS. A single-center study reported that argatroban was effective at decreasing the END rate and improving 7 days NIHSS scores, and also significantly improved the mRS score at 90 days within 48 h of stroke onset in patients with acute non-cardioembolic stroke not receiving IVT or EVT. Further subgroup analysis showed that for MIS, patients in the argatroban group had better clinical outcomes and lower END rates, although there was no significant difference in the 7 days NIHSS score between the argatroban group and the control group without argatroban (22). Another study found that compared with DAPT alone, DAPT plus argatroban reduced END occurrence and improved functional outcomes in branch atheromatous disease without increasing the risk of bleeding (23).

However, other studies have shown that argatroban combined with antiplatelet therapy has no advantage over antiplatelet monotherapy in improving neurological function in patients with AIS (24, 25). For patients with mild AIS, treatment regimens of argatroban combined with aspirin may be inferior to high-dose aspirin (aspirin 300 mg daily) in improving NIHSS scores (26). In patients with AIS within 4.5 h of symptom onset who received intravenous thrombolysis of alteplase, the combination of intravenous argatroban (100 μg/kg argatroban for 3 to 5 min within 1 h of the alteplase administered, followed by an argatroban infusion of 1.0 μg/kg per minute for 48 h) did not improve 90 days outcomes (27). Our study also showed that within 24 h after MIS onset, aspirin combined with argatroban (before the target APTT was taken into account) was not superior to DAPT in improving the END rate within 7 days.

Argatroban is short-acting and its anticoagulant actions can be monitored easily using tests such as activated clotting time and APTT (28). Currently, APTT is more commonly recommended for monitoring argatroban anticoagulation, with therapeutic anticoagulation being defined as an APTT value between 1.5- and 3-fold that at baseline and not exceeding 100 s (29, 30). Indeed, a proportion of stroke or TIA was the presenting manifestation of a haematologic disorder (31). Anticoagulant therapy may be more appropriate for stroke caused by hypercoagulable haematologic disorders. Cardiovascular risk factors, clinical features and early outcome of cerebral lacunar infarcts were also different from nonlacunar infarction (32). We speculate that the variation in the action of argatroban may be related to the differences in AIS pathogenesis, the level of APTT during treatment, and other factors. In a mouse model of carotid thrombosis, argatroban-treated animals with elevated levels of anticoagulation (2- to 3-fold the baseline APTT) exhibited an improved rate of rtPA-mediated recanalization (from 30% up to 45%) and a 2-fold improvement in carotid artery patency where recanalization occurred compared with rtPA therapy alone (33). In the ARTSS-2 trial, patients with AIS who were given a high-dose intravenous infusion of argatroban (100 μg/kg for 3–5 min within 1 h after rtPA followed by the continuous infusion with different doses of argatroban for 48 h) were divided into low-dose and high-dose groups adjusted to an APTT 1.75- and 2.25-fold that of baseline. It was found that the probability that combining argatroban with rtPA was superior to rtPA alone for the low, high, and low or high doses was 67, 74, and 79%, respectively (34).

In our study, after balancing baseline data with PSM, we also compared patients in the argatroban subgroup (those who achieved the target APTT in the argatroban group) with those in the DAPT group and found that the proportion of END and favorable functional outcomes at 7 days in the former group were superior to those in the latter group, which suggests that there is some clinical value in treating MIS based on the target APTT. However, there was no difference in long-term functional outcomes between the two groups. We further compared the baseline data between patients with and without END in the argatroban group using univariate analysis and found statistical differences in multiple baseline data, including the proportion of patients achieving the target APTT. However, after adjusting for confounding factors, we did not find that achieving the target APTT was an independent protective factor for END. It is unknown whether this is related to inappropriate target APTT setting or failure to maintain a continue target APTT.

Argatroban has shown promising results in safety studies. A systematic study showed an increase in systemic minor bleeding compared to placebo only at a high dose of argatroban [3 μg/(kg min^−1^)] (10). Another meta-analysis of 11 studies did not identify an increase in symptomatic intracranial hemorrhage, asymptomatic intracranial hemorrhage, gastrointestinal hemorrhage, systemic hemorrhage, or mortality, and only minor systemic hemorrhage (20). Our study also showed that there was no significant difference in the incidence of hemorrhage between the DAPT group and the argatroban group or the argatroban subgroup, and no symptomatic intracerebral hemorrhage occurred. Moreover, all the patients who experienced bleeding events did not require interruption of argatroban therapy after corresponding measures, including treatment with proton pump inhibitors, normal saline, bladder irrigation, and local compression, among other measures.

Our study had some limitations. First, this was a single-center, retrospective, observational study, which may produce assessment bias. Secondly, APTT was not continuously monitored during the use of argatroban. Again, we did not investigate the efficacy of agatroban in different pathogenesis or different types of stroke.

In conclusion, our findings suggested that, compared with DAPT, argatroban combined with aspirin, along with APTT monitoring, has some advantages in mitigating END in patients with MIS and improving their short-term functional prognosis without increasing the incidence of hemorrhage. However, whether or not an APTT level between 1.5 and 3 times that at baseline is a reasonable APTT target, and whether there are differences in the efficacy of agatroban for different pathogenesis and types of cerebral infarction, such as lacunar or non-lacunar, merits further investigation.

## Data availability statement

The raw data supporting the conclusions of this article will be made available by the authors, without undue reservation.

## Ethics statement

The studies involving humans were approved by the Ethics Committee of Suzhou Municipal Hospital. The studies were conducted in accordance with the local legislation and institutional requirements. The participants provided their written informed consent to participate in this study. Written informed consent was obtained from the individual(s) for the publication of any potentially identifiable images or data included in this article.

## Author contributions

XJ: Formal analysis, Funding acquisition, Writing – original draft, Writing – review & editing. XL: Data curation, Writing – original draft. HZ: Data curation, Writing – original draft. XY: Data curation, Writing – original draft. YG: Data curation, Writing – original draft. SP: Funding acquisition, Supervision, Writing – review & editing. LH: Formal analysis, Writing – original draft.
